# Effects of changes in straw chemical properties and alkaline soils on bacterial communities engaged in straw decomposition at different temperatures

**DOI:** 10.1038/srep22186

**Published:** 2016-02-26

**Authors:** Guixiang Zhou, Jiabao Zhang, Congzhi Zhang, Youzhi Feng, Lin Chen, Zhenghong Yu, Xiuli Xin, Bingzi Zhao

**Affiliations:** 1 State Key Laboratory of Soil and Sustainable Agriculture, Institute of Soil Science, Chinese Academy of Sciences, Nanjing 210008, China; 2 Poyang Lake Eco-economy Research Center, Jiujiang University, Jiujiang 332005, China; 3 University of Chinese Academy of Sciences, Beijing 100049, China; 4 Institute of Soil and Water Resources and Environmental Science, College of Environmental& Resource Sciences, Zhejiang University, Hangzhou 310058, China

## Abstract

Differences in the composition of a bacterial community engaged in decomposing wheat straw in a fluvo-aquic soil at 15 °C, 25 °C, and 35 °C were identified using barcode pyrosequencing. Functional carbon groups in the decomposing wheat straw were evaluated by ^13^C-NMR (nuclear magnetic resonance). *Actinobacteria* and *Firmicutes* were more abundant, whereas *Alphaproteobacteria* and *Bacteroidetes* were less abundant, at higher temperatures during the later stages of decomposition. Differences in the chemical properties of straw accounted for 19.3% of the variation in the community composition, whereas soil properties accounted for more (24.0%) and temperature, for less (7.4%). Carbon content of the soil microbial biomass and nitrogen content of straw were significantly correlated with the abundance of *Alphaproteobacteria, Actinobacteria*, and *Bacteroidetes*. The chemical properties of straw, especially the NCH/OCH_3_, alkyl O-C-O, and O-alkyl functional groups, exercised a significant effect on the composition of the bacterial community at different temperatures during decomposition—results that extend our understanding of bacterial communities associated with the decomposition of straw in agro-ecosystems and of the effects of temperature and chemical properties of the decomposing straw and soil on such communities.

The quantity and quality of soil organic matter (SOM) play a vital role in the sustainability of terrestrial ecosystems^1^,^2^,^3^. Large amounts of SOM are derived from plant residues. The decomposition of plant litter regulates the size of the soil carbon (C) pools and the recycling of plant nutrients, and also controls the CO_2_ fluxes to the atmosphere in terrestrial ecosystems^4^,^5^. The main organic components in plant litter are hydrophilic compounds, carbohydrate polymers (cellulose and hemicellulose), and lignin. Cellulose and hemicellulose are labile components in plant litter, whereas lignin—a complex polymer of aromatic rings—is a stable component that resists degradation^6^. The labile components are eventually exhausted, whereas the recalcitrant components tend to accumulate during decomposition, and these changes in the chemical composition of plant litter, including straw, regulate the composition of microbial communities engaged in decomposing the litter.

Recent studies suggest that microorganisms play a crucial role in the decomposition of plant litter in terrestrial ecosystems. For example, Pankratov *et al.*^7^ reported that bacteria are one of the main cellulose decomposers in *Sphagnum* peat, and Štursová *et al.*^8^ demonstrated that bacteria incorporated more ^13^C-labelled cellulose than fungi did during the decomposition of a layer of plant litter in a *Picea abies* forest in Europe, indicating that bacteria in the layer of plant litter are more active than fungi in the process of decomposition. Šnajdr *et al.*^9^ found that *Actinobacteria* play a key role in the C cycle and turnover of organic matter at later stages of litter decomposition. *Acidobacteria* is also considered to play an important role in degrading complex polymers such as cellulose and xylan^10^,^11^. However, the pattern of succession in a bacterial community engaged in decomposing straw with changes in its chemical properties at different stages of decomposition remains unclear.

Many studies have investigated the factors that influence the composition of microbial communities involved in decomposing straw. These factors include temperature, soil properties, and chemical composition of the litter^3^,^12^. Temperature, by changing the composition of the microbial community and the activity of different taxa that make up the community^13^,^14^,^15^, changes the production and activity of extracellular enzymes that catalyse litter decomposition^16^,^17^. An earlier study demonstrated that the carbon/nitrogen (C/N) ratio in soil and total organic C are significantly correlated with the composition of bacterial communities^18^. The chemical composition of straw is also influenced by changes in temperature during decomposition: changes in C/N ratio and lignin, for example, may lead to changes in the succession pattern of microbial communities^3^,^9^,^19^. However, these studies mostly focused on only a *single* factor: research on the *combined effects* of chemical properties of soil and straw on the composition of a microbial community at different temperatures and on the interaction between community composition and the chemical properties of straw is somewhat limited.

With this background, we propose that (1) temperature and soil properties will change the composition of a microbial community engaged in decomposing straw at different stages of decomposition and (2) the chemical composition of straw and the composition of microbial community are linked, and the links are influenced by changes in temperature. To test these hypotheses, we studied the decomposition of wheat straw at three temperatures (15 °C, 25 °C, and 35 °C) and followed the changes in chemical composition of straw and in the composition of the microbial community 30 days (early stage)^12^ and 120 days (late stage)^20^ after starting the experiment, (1) to evaluate the relative contributions of temperature, soil properties, and those of straw, to changes in the bacterial community and (2) to test the relationship between the chemical properties of straw and the composition of the bacterial community at the three temperatures.

## Results

### Effect of temperature on properties of soil and straw

Over 120 days of decomposition, no significant differences were found in the soil C/N ratio and the levels of dissolved organic carbon (DOC) at the different temperatures, nor did temperature have any significant effect on the C content of straw at the early stage (day 30). However, at the late stage (day 120), the C content at 15 °C was significantly greater than that at 25 °C. Microbial biomass carbon (MBC) and pH tended to decrease with the increase in temperature, whereas dissolved organic nitrogen (DON) in soil, and N and phosphorus (P) contents of straw, tended to increase with the increase in temperature on day 30 and on day 120 (Table 1).

Temperature greatly affected the proportions of C functional groups in the total spectral area in wheat straw (Fig. S1). At higher temperatures, the proportions of NCH/OCH_3_ and aromatic C–C+/H tended to increase whereas those of O-alkyl and alkyl O-C-O tended to decrease (Table 2); on the other hand, the proportion of alkyl C was not significantly correlated with temperature.

### Bacterial community diversity at different temperatures

Taking all the wheat straw samples together, a total of 179 560 sequences were obtained (Fig. S2). The average number of sequences per sample was 9975 and the range was 1100 to 33 987; nearly all (99.5%) the sequences could be classified (Table S2). Phylogenetic diversity (PD), Chao1 index, Shannon index, and richness in terms of the number of operational taxonomic units (OTUs) were estimated for 1050 randomly selected sequences per sample. No significant differences in PD and Chao1 index were seen between L30 (which denotes low temperature (15 °C) and the early stage of decomposition, that is day 30) and M30 (moderate temperature (25 °C) and day 30) and in Shannon index and OTU richness between L30 and H30 (high temperature (35 °C) and day 30) (Table 3). Two more treatments, namely M120 (moderate temperature and late stage) and H120 (high temperature and late stage), were similar in terms of PD, Shannon and Chao1 indexes, and OTU richness (Table 3). Overall, temperature had no significant effect on the bacterial diversity indexes (Table 3).

### Changes in composition, phyla and groups at different temperatures

**Figure 1:**
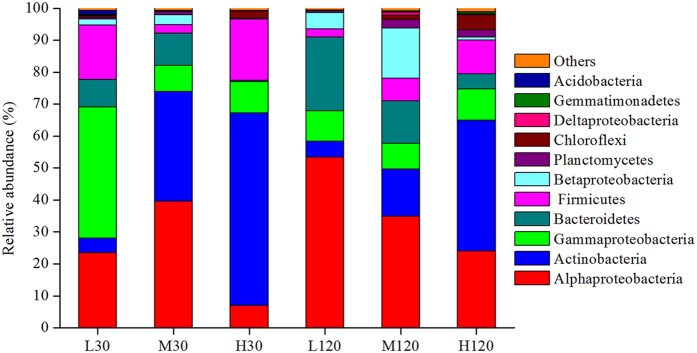
Relative abundance of dominant bacterial phyla in decomposing wheat straw. L30: low temperature (15 °C) on day 30 (early stage of decomposition); M30: moderate temperature (25 °C) on day 30; H30: high temperature (35 °C) on day 30; L120: low temperature on day 120 (late stage of decomposition); M120: moderate temperature on day 120; H120: high temperature on day 120.

Non-metric multidimensional scaling (NMDS) analysis clearly showed variations in the bacterial community among the different treatments (temperatures and the two stages of decomposition). The first axis differentiated microbial communities at 35 °C from those at 15 °C and 25 °C (Fig. S3), pointing to the clear effect of temperature. The dominant phyla across all the samples were *Alphaproteobacteria, Actinobacteria, Gammaproteobacteria, Bacteroidetes*, and *Firmicutes*, which together accounted for more than 78% of the sequences from each sample (Fig. 1). *Betaproteobacteria, Planctomycetes, Chloroflexi, Deltaproteobacteria, Gemmatimonadetes*, and *Acidobacteria* were also found in all the samples but in fairly small numbers (Fig. 1).

Temperature exerted highly specific effects on the relative abundance of different phyla (Fig. 1) in four distinct ways: (1) increase in relative abundance with increase in temperature on day 30 and day 120 (e.g. *Actinobacteria, P* < 0.001), (2) decrease in relative abundance with increase in temperature on day 120 (e.g. *Alphaproteobacteria* and *Bacteroidetes, P* < 0.001), (3) the lowest abundance (e.g. *Firmicutes, P* < 0.001) or the highest abundance (e.g. *Alphaproteobacteria, P* < 0.001) at moderate temperature on day 30, and (4) insensitivity to temperature (e.g. *Gammaproteobacteria, P* = 0.099).

**Figure 2:**
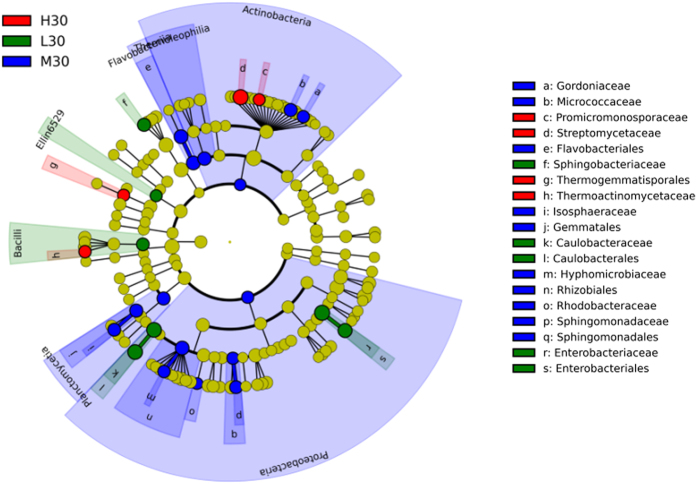
Composition of bacterial community at different temperatures on day 30 (early stage of decomposition of wheat straw). L30: low temperature (15 °C) on day 30 (early stage of decomposition); M30: moderate temperature (25 °C) on day 30; H30: high temperature (35 °C) on day 30.

**Figure 3:**
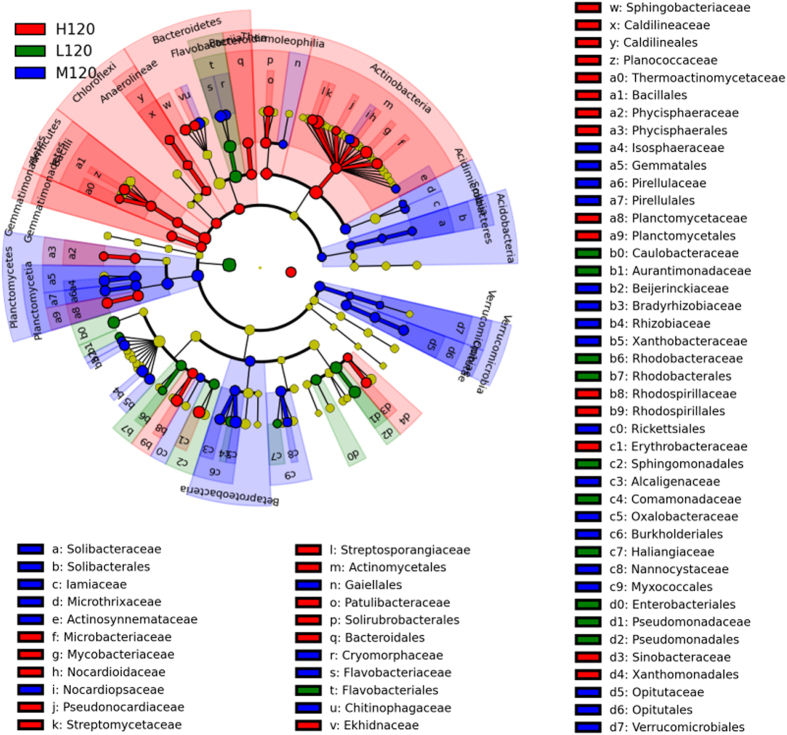
Composition of bacterial community at different temperature on day 120 (late stage of decomposition of wheat straw). L120: low temperature on day 120 (late stage of decomposition); M120: moderate temperature on day 120; H120: high temperature on day 120.

The taxa that differed between the different treatments were identified by linear discriminant effect size (LEfSe) analysis (Fig. 2 shows the early stage and Fig. 3 shows the late stage). Temperature was closely related to the abundance of different species, particularly *Actinobacteria* and *Proteobacteria*, on day 30. As shown by the circular cladogram, *Sphingobacteriaceae, Caulobacteraceae*, and *Enterobacteriaceae* were significantly more abundant in L30; *Gordoniaceae, Micrococcaceae, Isosphaerceae, Hyphomicrobiaceae, Rhodobacteraceae*, and *Sphingomonadaceae* in M30; and *Promicromonosporaceae, Streptomycetaceae*, and *Themoactinomycetaceae* in H30 (Fig. 2). The richness and diversity of some microbial groups were greater on day 120 than on day 30 (Fig. 3): *Caulobacteraceae, Comamonadaceae*, and *Pseudomonadaceae* were more abundant in L120; *Cryomorphaceae, Flavobacteriaceae, Isosphaeraceae, Pirellulaceae, Rhizobiaceae*, and *Xanthobacteraceae* in M120; and *Mycobacteriaceae, Streptomycetaceae, Rhodospirillaceae*, and *Erythrobacteraceae* in H120 (Fig. 3).

**Figure 4:**
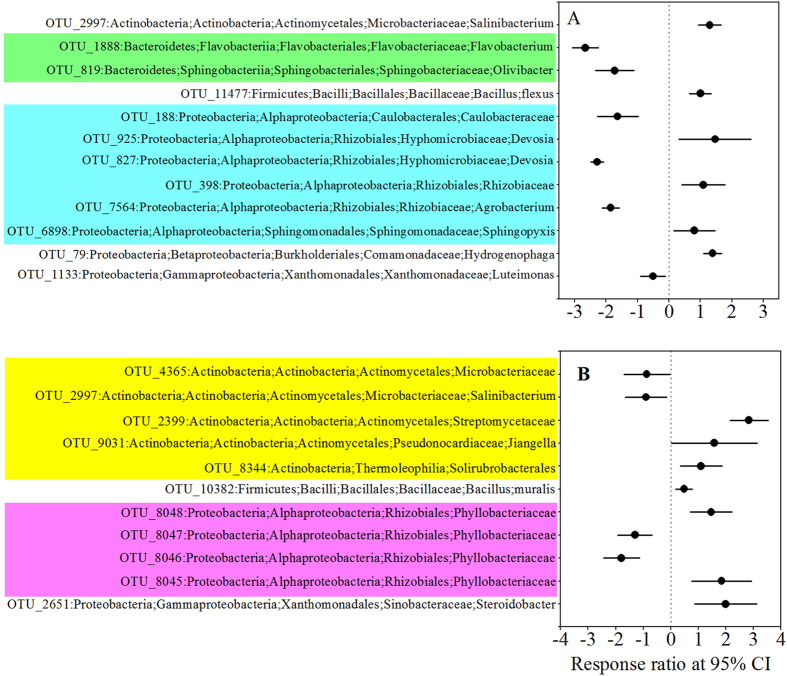
Operational taxonomic units that showed significant changes in abundance at 25 °C as compared to that at 15 °C (**A**) and at 35 °C (**B**) on day 120 (late stage of decomposition of wheat straw). Significance was determined using response ratio methods at a confidence interval of 95%.

Several phyla responded inconsistently to temperature (Fig. 4). The abundance of *Actinobacteria* and *Betaproteobacteria* increased significantly from 15 °C to 25 °C, whereas that of *Bacteroidetes* and *Gammaproteobacteria* decreased significantly (Fig. 4A); on the other hand, *Alphaproteobacteria* (phylum *Proteobacteria*) remained unaffected. Similar patterns were observed between 25 °C and 35 °C except that *Actinobacteria* showed no significant difference and *Gammaproteobacteria* was significantly more abundant at 35 °C (Fig. 4B).

### Changes in composition and phyla due to changes in properties of soil and straw

**Figure 5:**
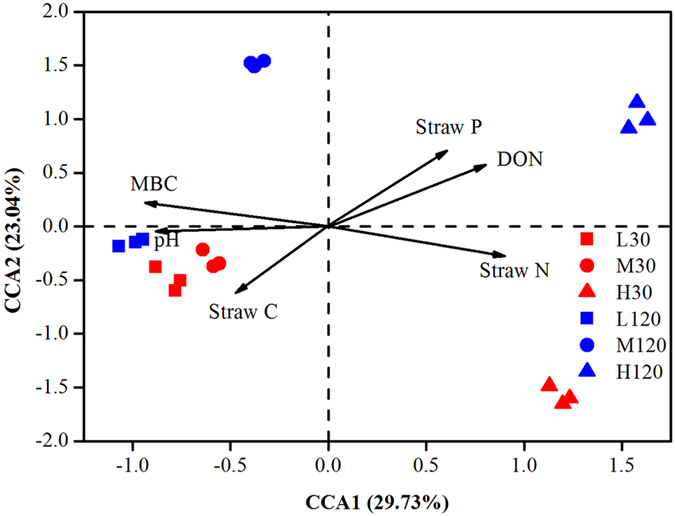
Canonical correspondence analysis (CCA) of bacterial communities under different treatments. L30: low temperature (15 °C) on day 30 (early stage of decomposition); M30: moderate temperature (25 °C) on day 30; H30: high temperature (35 °C) on day 30; L120: low temperature on day 120 (late stage of decomposition); M120: moderate temperature on day 120; H120: high temperature on day 120.

**Figure 6:**
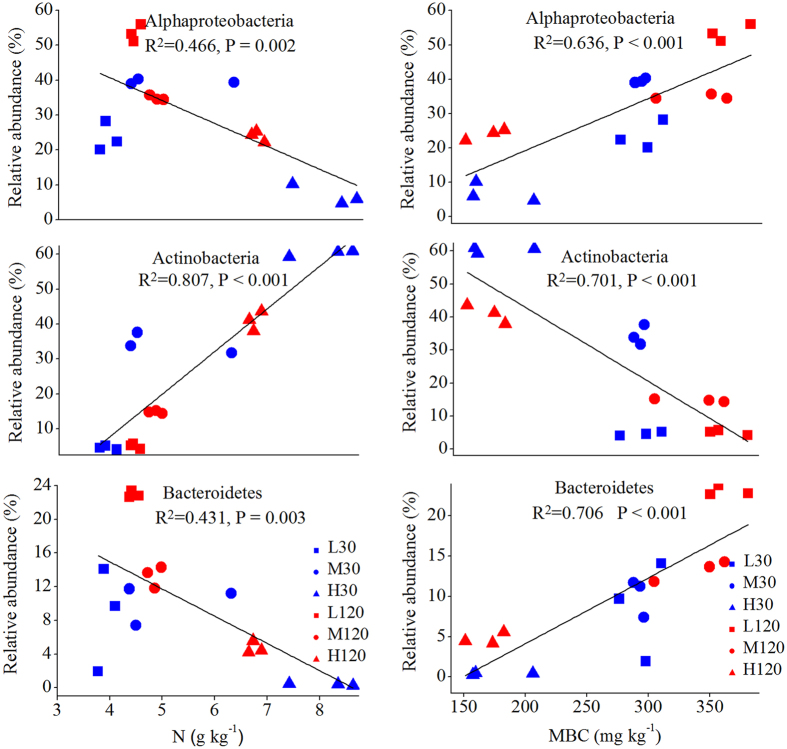
The relationship between abundance of dominant bacterial groups and (1) nitrogen content of straw and (2) soil microbial biomass carbon (MBC) contents under different treatments. Linear regressions were used to test the correlation.

**Figure 7:**
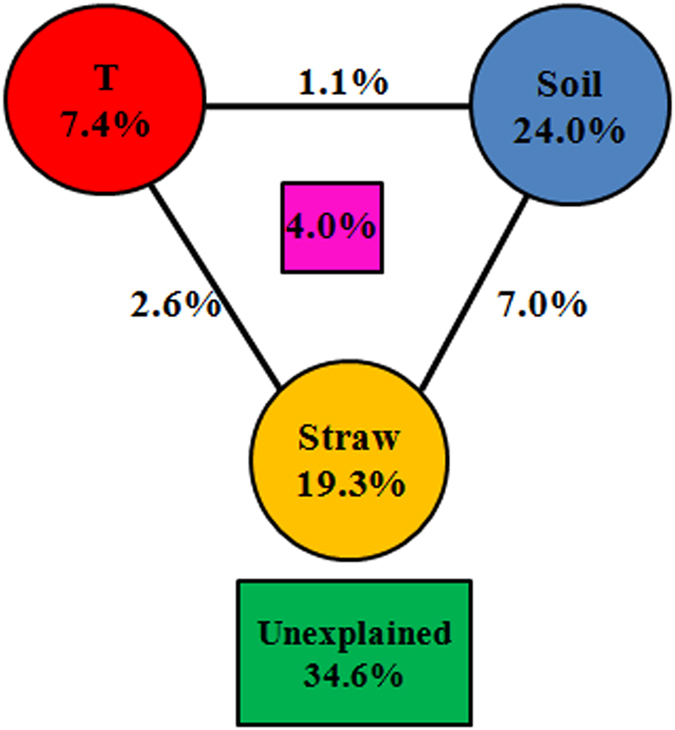
Variation partitioning analysis (VPA) of the effects of temperature, soil properties, and properties of straw on composition of bacterial community. T = temperature.

Mantel tests showed that the composition of the bacterial community was significantly correlated with such properties of soil and straw as pH, MBC, and N content (Table S3). Among the soil properties, the correlation was particularly strong for pH, DON, and MBC (*P* = 0.001) and among the properties of straw, for levels of C, N, and P (*P* < 0.01) (Table S3). Factors that influenced the composition of the bacterial community significantly were chosen for canonical correspondence analysis (CCA), which showed that soil MBC and straw N content exerted particularly strong effects (Fig. 5). These results were similar to those of the Mantel test. The effects of MBC and straw N content on the dominant phyla were examined using linear regression analysis: it is noteworthy that soil MBC was significantly (*P* < 0.001) correlated with the abundance of *Alphaproteobacteria, Actinobacteria*, and *Bacteroidetes*; the N content was also significantly (*P* < 0.01) correlated with the same three phyla, but the direction of the effect was opposite of that in the case of MBC (Fig. 6). Variation partitioning analysis (VPA) showed that combinations of temperature, soil properties (including pH, DOC, DON, and MBC), and the properties of straw (total C, N, P) were significantly (*P* = 0.002) correlated with the community composition (pairwise Jaccard distances between samples) (Fig. 7). These variables explained 65.4% of the observed variation; the rest remained unexplained. Among the factors mentioned above, soil properties explained 24.0% (*P* = 0.002) of the variation; temperature, 7.4% (*P* = 0.014); the properties of straw, 19.3% (*P* = 0.007); and the interaction among the three variables, 4.0% (Fig. 7). Path analysis demonstrated that the direct effect of temperature on bacterial community composition was less than the effects of soil and straw properties (Table S4). Namely, the indirect effects of temperature via changes in soil and straw properties are more significant than its direct effect on bacterial composition.

**Figure 8:**
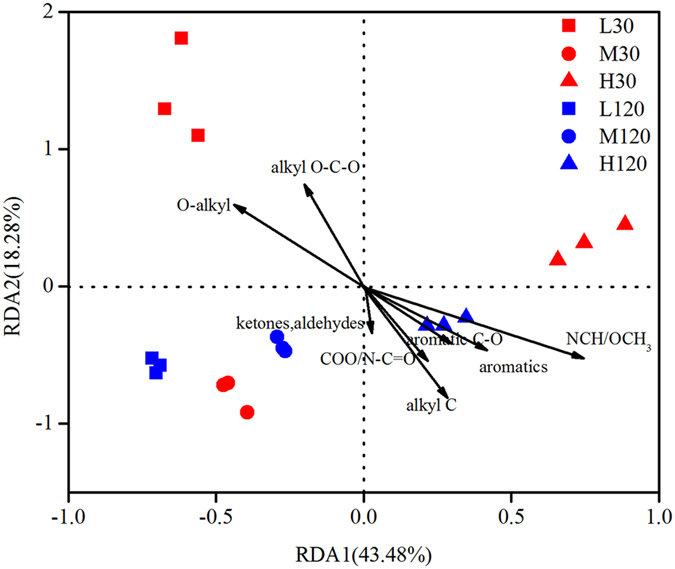
Redundancy analysis (RDA) relating bacterial community composition to straw quality during decomposition. L30: low temperature (15 °C) on day 30 (early stage of decomposition); M30: moderate temperature (25 °C) on day 30; H30: high temperature (35 °C) on day 30; L120: low temperature on day 120 (late stage of decomposition); M120: moderate temperature on day 120; H120: high temperature on day 120.

Changes in the availability of C—particularly that of easily water-soluble C—change the composition of the microbial community. However, which compounds regulate the community dynamics in later stages of decomposition once the easily available C is depleted? An earlier study indicated that 30 days is not long enough to detect changes in the chemistry of the residue because decomposition is slow in the first few days^21^. Therefore, functional groups in straw that had been decomposing for 120 days rather than for 30 days were taken in the present experiment for redundancy analysis (RDA). Axis 1 separated O-alkyl and alkyl O-C-O groups from the other functional groups and explained 43.48% of the variation, whereas axis 2 explained 18.28% of the variation. The biplots of functional groups of C from samples of straw indicated that NCH/OCH_3_ (45–60 ppm) and O-alkyl (60–93 ppm) had greater effects (the longer arrow) on the composition of the bacterial community (Fig. 8).

## Discussion

We found that indirect instead of direct effects of temperature via soil and chemical properties of straw influence microbial community. Temperature explained only 7.4% of the variation in the composition of the bacterial community, whereas the soil and straw variables explained as much as 24.0% and 19.3%, respectively (Fig. 7). Path analysis also demonstrated that the direct effects of higher temperature on bacterial communities were minor relative to the potential indirect effects (Table S4). A similarly minor effect of higher temperatures on bacterial communities compared to that of soil and plant variables was reported in a field experiment on an alpine meadow in a Tibetan plateau^22^. Our study clarified that the indirect effects of temperature, which contained the changes of C, N availability and chemical groups of straw, played an important role in the decomposing bacterial community structure during wheat straw decomposition.

### The changes in C availability influence the decomposing bacterial community

The present experiment clearly showed the effects of temperature on the structure of bacterial communities (Table 3, Figs 2 and 3 and Fig. S3)—effects that are consistent with those reported earlier^13^,^23^,^24^ and with our first hypothesis. Plant straw is very rich in carbon and nitrogen, as well as containing an abundance of certain more readily degradable molecules such as cellulose and hemicellulose^5^. *Alphaproteobacteria, Actinobacteria* and *Bacteroidetes* have been known to degrade organic materials and plant-derived polymers^5^ and became more abundant in response to straw returning. The abundances of *Alphaproteobacteria, Actinobacteria* and *Bacteroidetes*, which are considered to have a positive correlation with soil available C^25^, varied with the temperatures. Stark *et al.*^15^ observed that *Alphaproteobacteria* became more abundant as soil temperature increased from 4 °C to 14 °C. Yergeau *et al.*^13^ also found that *Alphaproteobacteria* and *Actinobacteria* were more abundant in warmer soils (warmer by 0.5–2 °C), probably because more soil organic C was available in those soils, which, in turn, favoured the fast-growing microorganisms (the copiotrophs, such as *Alphaproteobacteria* and *Bacteroidetes*) over the oligotrophs such as *Actinobacteria*^26^. However, the present study showed the opposite response: *Alphaproteobacteria* were *less* abundant at 35 °C than at 15 °C on day 120 (Fig. 1). The much greater increase in temperature—from 15 °C to 35 °C—in the present experiment may have depleted soil organic C, thereby limiting the reproduction of copiotrophs and favouring that of oligotrophs instead. Thus, temperature-induced changes in bacterial community may, in turn, regulate the characteristics of soil and of straw, ultimately changing the C cycles in terrestrial ecosystems^27^,^28^,^29^,^30^,^31^.

### The change in N availability during straw decomposition influences bacterial community structure

An increase in the N content of straw was observed with time due to the accumulation of N from the more recalcitrant compounds of N, which, by definition, take longer to decompose. Over time, as the litter quantity and/or quality decreased, *Actinobacteria* and *Bacteroidetes* became more abundant. *Actinobacteria*, which are oligotrophs, play a crucial role in degrading recalcitrant compounds such as cellulose and xylan^5^,^10^. *Bacteroidetes* is also considered to compete well when the availability of substrate is low^3^ and possesses a great capacity for degrading carbohydrates^7^. These results indicate a link between the attributes of straw, such as its N content, and the community of bacteria engaged in decomposing the straw. Straw N content showed a significant linear correlation with the dominant phylum (Fig. 6). Cleveland *et al.*^4^ found that litter quality explained 16% of the variation in the composition of a bacterial community, and in the present experiment, properties of straw explained 19.3% of such variation (Fig. 7). Microorganisms use available C in the straw in the initial stages of decomposition; in its later stages, they use N, accumulated, by that time, as a result of the breakdown of recalcitrant N compounds^32^. The quantity and quality of straw as a substrate, especially its N contents, regulate the bacterial communities (Fig. 5), probably because the easily available N compounds favour copiotrophs such as *Alphaproteobacteria* and *Bacteroidetes* in the early stages of decomposition, whereas the recalcitrant N compounds favour oligotrophs such as *Actinobacteria* in the later stages.

### The changes in chemical groups of straw influence the decomposing bacterial community

Redundancy analysis based on ^13^C chemical groups of straw to evaluate more precisely the effect of changes in chemical properties of straw on the structure of the bacterial community showed that changes in community composition were related mainly to the concentrations of NCH/OCH_3_ (45–60 ppm) and O-alkyl (60–93 ppm) (Fig. 8). The concentration of alkyl O-C-O, found mainly in carbohydrates, was also significantly (*P* < 0.05) correlated to the diversity of the microbial community. However, carbohydrates such as sugars, which were dominant in the early stage, were depleted in the later stage of decomposition, and the effect of alkyl O-C-O was weaker than that of NCH/OCH_3_ and O-alkyl at the later stage: NCH/OCH_3_ is found mainly in nucleotides, proteins, and lignin, which are more resistant to decomposition than carbohydrates are. Aromatics and alkyl C, found mainly in the lignin in wheat straw^33^, also affected the composition of the microbial community (Fig. 8), indicating its strong link with the lignin content of straw. These results support our second hypothesis, namely that chemical properties of straw are strongly correlated with the structure of the bacterial community that decomposes the straw.

Earlier studies were focused on the effects of temperature *either* on the chemical properties of straw and its decomposition^34^,^35^ *or* on the microbial community during the decomposition of straw^3^,^36^: the *interaction* between straw and the microbial community at different temperatures was most often ignored—the present study, however, shows a clear link between the two (Fig. 8). Baumann *et al.*^33^, using different types of residue, investigated the relationship between residual C and composition of the microbial community by means of phospholipid fatty acid (PLFA) analysis and found that the differences in the proportions of alkyl O-C-O were strongly related to the composition of the microbial community irrespective of the type of residue (eucalyptus, wheat, and vetch), although in the case of the eucalyptus residue, O-alkyl C played a more important role than alkyl O-C-O did. In the present study, the proportion of alkyl O-C-O, found mainly in carbohydrates such as sugars, decreased at the higher temperatures (Table 2), suggesting that the differences in the microbial community reflect the differences in soluble sugars. The ^13^C chemical groups of O-alkyl moieties (60–93 ppm), which are attributed mainly to C2-C6 groups of carbohydrates^37^ and lignin, were preferentially degraded, and the proportion of O-alkyl decreased at the higher temperatures (Table 2). These results suggest that both carbohydrates and lignin influenced succession in the microbial community at different temperatures. In addition, NCH/OCH_3_ was also associated with the differences in the community structure: NCH, which is attributed to proteins or peptides, increased at the higher temperature, as did OCH_3_, which is attributed to lignin^37^. These data are in agreement with those of the study mentioned above^33^, indicating that proteins or amino acids are important drivers of the structure of microbial communities engaged in decomposing the residues of organic matter in soil.

## Methods

### Soil collection

One soil sample was collected from the Fengqiu Agro-Ecological Experimental Station of the Chinese Academy of Sciences, Fengqiu County (35°00′ N, 114°24′ E), Henan province, China. The annual average precipitation is this region is 615 mm and the average temperature is 13.9 °C. The soil is sandy loams (40.7% silt, 45.6% sand, and 13.7% clay), derived from the alluvial sediment of the Yellow River, and classified as Aquic Inceptisols according to the USDA classification system. The soil sample was taken from the plough layer (0–20 cm) after the harvest of wheat in May 2013 and sieved through a 2 mm sieve after removing visible plant litter and stones. The soil sample was divided into two subsamples, one was air-dried and the other was stored at 4 °C.

### Straw incorporation and soil incubation

Wheat (*Triticum aestivum* L.) straw was sampled from the same site in May 2013, oven-dried at 60 °C, and cut into 2 cm lengths. The straw samples were placed in double-layered nylon bags (10 cm × 10 cm, 0.074 mm mesh) to prevent the straw from being mixed with the surrounding soil. Each bag contained 10 g of wheat straw and was buried horizontally in 2 L plastic jars, each filled with 400 g of air-dried soil. The jars were incubated at 15 °C, 25 °C, and 35 °C for 120 days. Each treatment had three replicates. Soil moisture content was maintained at 70% of the maximum water-holding capacity (WHC, w/w) by adding distilled water whenever required. Three nylon mesh bags at each temperature were collected after 30 days and three more after 120 days and the samples were weighed: one subsample was oven-dried to determine the moisture content and the other stored at −20 °C.

### Chemical and biological analyses of soil

Soil pH was measured with a pH meter using soil suspension (1 part of soil and 5 parts of water, w/v). Soil total C (TC) was determined by dichromate oxidation and total nitrogen (TN) by titration with ferrous ammonium sulphate^38^; DOC and DON were extracted with 2M KCl and were analysed using a TOC analyser (Multi N/C 3100, Analytik Jena, Germany); and MBC and microbial biomass N (MBN) were analysed by the chloroform fumigation and extraction method^39^. The results were calculated using 0.38 (K_C_) and 0.45 (K_N_) as correction factors^40^ for C and N, respectively. For the straw, TC was determined by dichromate oxidation; TN, by the Kjeldahl method; and TP, by spectrophotometry^34^,^41^.

### ^13^C-NMR Spectroscopy

The straw samples were ground fine enough to pass through a 0.074 mm sieve for NMR analysis. Information on the functional groups of C in the straw was obtained by solid-state ^13^C NMR analysis using a Bruker Avance 400 spectrophotometer (BrukerBioSpin, Rheinstetten, Germany) at a frequency of 100.6 MHz for ^13^C. Approximately 100 mg of straw was packed in a 4 mm sample rotor and spun at 5 kHz. The contact time and the recycle delays were 1 ms and 2 s, respectively. The number of scans was 2000 for each sample.

The spectra were integrated into eight chemical-shift regions, with assignments as follows^42^,^43^: 0–45 ppm, nonpolar alkyl; 45–60 ppm, NCH/OCH_3_; 60–93 ppm, O–alkyl; 93–110 ppm, alkyl O–C–O; 110–142 ppm, aromatic C–C + /H; 142–165 ppm, aromatic C–O; 165–190 ppm, COO/N–C = O, and 190–220 ppm, ketones or aldehydes.

### Extraction of DNA

Straw residues (0.1 g per sample) were homogenized under sterile conditions, and total DNA was extracted using a FastDNA SPIN kit (MP Biomedicals, Santa Ana, California, USA) following the procedure recommended by the manufacturer. The extracted DNA was dissolved in 50 mL TE buffer, quantified with a NanoDrop ND-1000 spectrophotometer (NanoDrop Technologies, Wilmington, Delaware, USA), and stored at −20 °C until required.

### 16S rRNA gene amplification for 454 sequencing

The amplification of 16S rRNA genes for 454 sequencing was performed as described elsewhere in detail^44^ and briefly as follows: an aliquot (50 ng) of purified DNA from each straw sample was used as a template for PCR amplification. The primer set F515 (5ʹ-GTGCCAGCMGCCGCGG-3ʹ) with the Roche 454 ‘A’ pyrosequencing adapter and R907 (5ʹ-CCGTCAATTCMTTTRAGTTT-3ʹ) with the Roche 454 ‘B’ sequencing adapter at the 5ʹ-end of each primer were used to amplify the V4-V5 region of the bacterial 16S rRNA genes. The targeted gene region is considered the most appropriate for accurate phylogenetic reconstruction of bacteria^45^. A unique 7-bp barcode sequence was inserted into the primers to distinguish the amplified products (Table S1). The reaction was performed using a total of 50 μL of each sample in triplicate under following conditions: 94 °C for 5 min; 30 cycles at 94 °C for 30 s, at 55 °C for 30 s, and at 72 °C for 30 s; and the final extension at 72 °C for 10 min. The products of the PCR were pooled and purified using an agarose gel DNA purification kit (TaKaRa Bio, Tokyo, Japan). Equal amounts of the PCR products of each sample were quantified by NanoDrop and combined in a single tube to be run on a Roche FLX 454 pyrosequencing machine (Roche Diagnostics Corporation, Branford, Connecticut, USA).

### Analysis of the 454 pyrosequencing data

The pyrosequencing data were processed using the software package Quantitative Insights Into Microbial Ecology (QIIME) (ver. 1.8.0)^46^. Reads shorter than 200 bp or longer than 400 bp were discarded. Homopolymers longer than 6 bp were eliminated by PyroNoise^47^. Bacterial phylotypes were identified using UCLUST (Edgar, 2010), and high-quality sequences were clustered into OTUs with 97% similarity. The most abundant sequence for each OUT was selected as the representative sequence, which was aligned using PyNAST^48^. The taxonomic identity of each phylotype was determined using the ribosomal database project (RDP) Classifier^49^. To correct for survey effort, a randomly selected subset of 1050 sequences per sample was used for comparing the differences between samples. Finally, the OTUs were used in the LEfSe linear discriminant analysis (LDA) coupled with effect-size measurements to characterize the taxonomic guilds most distinctly responding to different temperatures.

### Statistical analysis

Four metrics were used for assessing the bacterial alpha-diversity: phylogenetic diversity (PD)^50^, the Shannon index^51^, the Chao1 index^52^, and the observed OTU richness. A one-way ANOVA was used to test for significant differences (*P* < 0.05) across the treatments according to Tukey’s test and linear regression analyses were used for testing the relationships between the taxonomic diversity of a group and soil properties or the chemical properties of straw using SPSS ver. 17.0. Path analysis was used to evaluate the direct and indirect effects of pathways of responses relative to bacterial community composition using SPSS ver. 17.0. Mantel tests were used for identifying the factors that correlated significantly with community composition (abundance of OTUs) and were performed in the Vegan package of R ver. 2.8.1 (R Development Core Team. Vienna, Austria). Variance inflation factors (VIFs) were used for ascertaining whether the correlation between environmental factors and community composition was significant. Canonical correspondence analysis was performed with the VIFs, the value of which was less than 20. Non-metric multidimensional scaling was used for evaluating overall differences within the community composition based on pairwise Jaccard distances, and RDA was used for assessing the relationship between community composition and the chemical properties of straw. The analyses, namely CCA, CCA-based variation partitioning analysis (VPA), NMDS, and RDA, were completed using the Vegan package of R ver. 2.8.1.

## Additional Information

**How to cite this article**: Zhou, G. *et al.* Effects of changes in straw chemical properties and alkaline soils on bacterial communities engaged in straw decomposition at different temperatures. *Sci. Rep.* **6**, 22186; doi: 10.1038/srep22186 (2016).

## Supplementary Material

Supplementary Information

## Figures and Tables

**Table 1:** Chemical properties of soil and wheat straw at early and late stages of decomposition at different temperatures.

Parameter	L30	M30	H30	L120	M120	H120
pH	7.42 ± 0.02a	7.38 ± 0.01ab	7.27 ± 0.01bc	7.47 ± 0.02a	7.36 ± 0.05ab	7.24 ± 0.01c
C/N ratio	20.03 ± 0.53a	19.44 ± 0.45a	19.43 ± 0.58a	18.12 ± 0.12a	18.11 ± 0.60a	17.41 ± 0.87a
DOC (mg kg^–1^)	125.44 ± 1.92a	126.19 ± 9.40a	130.40 ± 4.04a	118.85 ± 2.76a	117.62 ± 2.87a	134.73 ± 4.71a
DON (mg kg^–1^)	81.57 ± 3.09d	99.95 ± 2.83d	148.96 ± 1.74c	70.05 ± 4.86d	185.12 ± 6.58b	234.85 ± 12.77a
MBC (mg kg^–1^)	295.15 ± 10.01b	292.53 ± 2.46b	174.51 ± 15.91c	362.98 ± 9.34a	338.69 ± 17.35ab	169.14 ± 9.29c
MBN (mg kg^–1^)	21.08 ± 4.11c	35.52 ± 2.70abc	46.19 ± 3.09ab	34.36 ± 5.54bc	52.61 ± 4.31a	39.55 ± 1.19ab
Straw C (g kg^–1^)	414.85 ± 12.31bc	434.30 ± 5.19b	418.14 ± 18.31b	490.63 ± 6.39a	342.40 ± 11.91d	361.68 ± 8.56cd
Straw N (g kg^–1^)	3.93 ± 0.10b	5.06 ± 0.63b	8.14 ± 0.37a	4.45 ± 0.05b	4.85 ± 0.07b	6.76 ± 0.07a
Straw P (g kg^–1^)	0.53 ± 0.01e	0.58 ± 0.03e	0.90 ± 0.05d	1.11 ± 0.05c	1.45 ± 0.01b	2.02 ± 0.02a

L30: low temperature (15 °C) on day 30 (early stage of decomposition); M30: moderate temperature (25 °C) on day 30; H30: high temperature (35 °C) on day 30; L120: low temperature on day 120 (late stage of decomposition); M120: moderate temperature on day 120; H120: high temperature on day 120.

C/N ratio: soil carbon/nitrogen ratio, DOC: soil dissolved organic carbon, DON: soil dissolved organic nitrogen, MBC: soil microbial biomass carbon, MBN: soil microbial biomass nitrogen.

The values are average values ± standard error (n = 3). Different letters within a row indicate a significant difference (*P* < 0.05) between treatments according to Tukey’s test.

**Table 2:** The assignment of functional groups at different chemical-shift regions and their proportions in the total spectral area determined by ^13^C NMR.

Group	190–220 ppm	165–190 ppm	142–165 ppm	110–142 ppm	93–110 ppm	60–93 ppm	45–60 ppm	0–45 ppm
Assignment	ketones/aldehydes	COO/N–C = O	aromatic C–O	aromatic C–C + /H	alkyl O–C–O	O–alkyl	NCH/OCH_3_	alkyl
L30	1.4 ± 0.2cd	6.2 ± 0.1d	3.9 ± 0.1d	9.4 ± 0.1c	9.8 ± 0.2a	42.8 ± 1.0a	8.4 ± 0.1d	18.2 ± 1.2a
M30	1.0 ± 0.1d	6.7 ± 0.1d	3.9 ± 0.0d	9.7 ± 0.1c	8.5 ± 0.1b	38.1 ± 0.1b	10.9 ± 0.1b	21.2 ± 0.1ab
H30	2.0 ± 0.4bc	8.7 ± 0.3c	5.5 ± 0.3c	11.9 ± 0.3b	7.9 ± 0.1b	30.7 ± 0.2c	11.9 ± 0.3a	21.5 ± 1.1ab
L120	2.9 ± 0.0ab	9.9 ± 0.1b	5.8 ± 0.1bc	11.7 ± 0.1b	7.3 ± 0.1c	31.1 ± 0.1c	9.8 ± 0.1c	21.5 ± 0.2a
M120	3.0 ± 0.1a	9.9 ± 0.1b	6.2 ± 0.0b	12.5 ± 0.1ab	7.0 ± 0.0d	30.2 ± 0.1c	9.9 ± 0.1c	21.2 ± 0.1ab
H120	3.0 ± 0.1ab	10.8 ± 0.2a	7.4 ± 0.1a	12.9 ± 0.2a	6.5 ± 0.0cd	24.8 ± 0.2d	11.7 ± 0.2a	23.0 ± 0.4a

L30: low temperature (15 °C) on day 30 (early stage of decomposition); M30: moderate temperature (25 °C) on day 30; H30: high temperature (35 °C) on day 30; L120: low temperature on day 120 (late stage of decomposition); M120: moderate temperature on day 120; H120: high temperature on day 120.

**Table 3:** Bacterial alpha-diversity in all treatments sampled on day 30 and day 120 of the incubation period.

Treatment	Bacterial alpha-diversity			
	PD	Shannon	Chao1	OTU richness
L30	20.9 ± 1.9bc	4.8 ± 0.3d	567.0 ± 47.9a	169.2 ± 20.3cd
M30	23.0 ± 0.3bc	6.1 ± 0.1bc	666.9 ± 61.0a	264.6 ± 5.9ab
H30	13.5 ± 1.3d	4.5 ± 0.2d	298.8 ± 15.5b	139.8 ± 8.0d
L120	19.8 ± 0.9c	5.5 ± 0.1c	559.6 ± 71.1a	208.6 ± 17.8bc
M120	29.0 ± 0.3a	7.0 ± 0.0a	684.7 ± 8.1a	298.7 ± 1.7a
H120	25.3 ± 0.3ab	6.4 ± 0.0ab	619.2 ± 20.3a	257.7 ± 2.8ab

L30: low temperature (15 °C) on day 30 (early stage of decomposition); M30: moderate temperature (25 °C) on day 30; H30: high temperature (35 °C) on day 30; L120: low temperature on day 120 (late stage of decomposition); M120: moderate temperature on day 120; H120: high temperature on day 120. PD, Shannon, Chao1, and OTU richness are phylogenetic diversity, Shannon index, Chaol index, and estimated maximum OTU number and observed OTU number, respectively. The diversity indexes were calculated using 1050 randomly selected sequences per sample. Results are expressed as mean ± standard error for the amended treatments (n = 3), and different letters within a column indicate a significant difference at *P* < 0.05 based on Tukey’s test.
