# Supplementary material for: Effects of changes in straw chemical properties and alkaline soils on bacterial communities engaged in straw decomposition at different temperatures
Source: Sci Rep. 2016 Feb 26;6:22186. doi: 10.1038/srep22186 (PMC4768159; doi:10.1038/srep22186)
Supplement: Supplementary Information [file srep22186-s1.pdf]

Supplementary information

Effects of changes in straw chemical properties and alkaline soils on bacterial communities engaged in straw decomposition at different temperatures

Guixiang Zhou[1,2,3], Jiabao Zhang[1*], Congzhi Zhang[1], Youzhi Feng[1], Lin Chen[4], Zhenghong Yu[1,3], Xiuli Xin[1], Bingzi Zhao[1]

[1]State Key Laboratory of Soil and Sustainable Agriculture, Institute of Soil Science, Chinese Academy of Sciences, Nanjing 210008, China

[2]Poyang Lake Eco-economy Research Center, Jiujiang University, Jiujiang 332005, China

[3]University of Chinese Academy of Sciences, Beijing 100049, China

[4]Institute of Soil and Water Resources and Environmental Science, College of Environmental& Resource Sciences, Zhejiang University, Hangzhou 310058, China

Correspondence and requests for materials should be addressed to J.-B. Zhang. (email: jbzhang@issas.ac.cn).

Figure S1. ^{13}C-NMR spectra of the decomposed wheat straw under different temperature and incubation time. L30: low temperature (15 ℃) on day 30 (early stage of decomposition); M30: moderate temperature (25 ℃) on day 30; H30: high temperature (35 ℃) on day 30; L120: low temperature on day 120 (late stage of decomposition); M120: moderate temperature on day 120; H120: high temperature on day 120.

Figure S2. The proportional distributions of sequences and derived OTUs in each bacterial phylum detected in all straw samples.

Figure S3. Non-metric multidimensional scaling (NMDS) of the bacterial communities based on Jaccard distances and symbols coded by treatments. L30: low temperature (15 ℃) on day 30 (early stage of decomposition); M30: moderate temperature (25 ℃) on day 30; H30: high temperature (35 ℃) on day 30; L120: low temperature on day 120 (late stage of decomposition); M120: moderate temperature on day 120; H120: high temperature on day 120.

Table S1. List of straw samples and corresponding barcodes used in this study. L30: low temperature (15 ℃) on day 30 (early stage of decomposition); M30: moderate temperature (25 ℃) on day 30; H30: high temperature (35 ℃) on day 30; L120: low temperature on day 120 (late stage of decomposition); M120: moderate temperature on day 120; H120: high temperature on day 120.

Table S2. Number of sequences and OTUs measured by QIIME processing in each sample. L30: low temperature (15 ℃) on day 30 (early stage of decomposition); M30: moderate temperature (25 ℃) on day 30; H30: high temperature (35 ℃) on day 30; L120: low temperature on day 120 (late stage of decomposition); M120: moderate temperature on day 120; H120: high temperature on day 120.

Table S3. The factors that significant correlated with bacterial communities of decomposing wheat straw were list below. The correlations (*r*) and significance (*P*) were determined by Mantel analysis between the variables and the bacterial community composition.

Table S4. Direct and indirect effects of pathways of responses relative to bacterial community by path analysis.

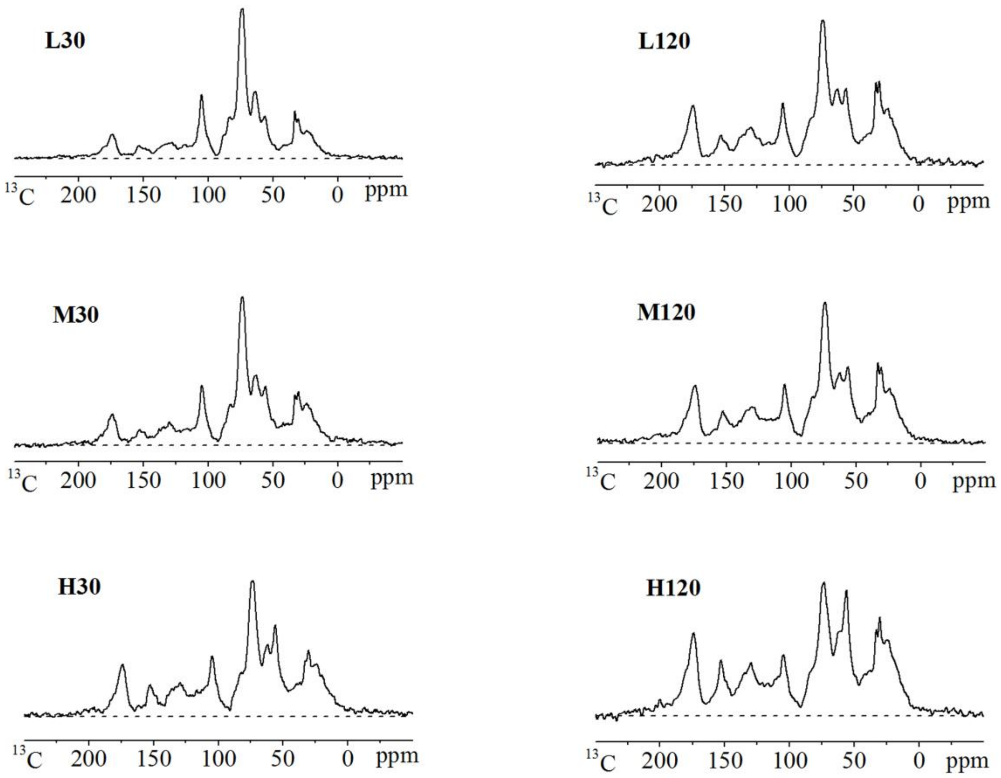

Figure S1. ^{13}C-NMR spectra of the decomposed wheat straw under different temperature and incubation time. L30: low temperature (15 ℃) on day 30 (early stage of decomposition); M30: moderate temperature (25 ℃) on day 30; H30: high temperature (35 ℃) on day 30; L120: low temperature on day 120 (late stage of decomposition); M120: moderate temperature on day 120; H120: high temperature on day 120.

Taxonomy	Sequences	OTUs
Alphaproteobacteria	76110	2251
Betaproteobacteria	13075	279
Deltaproteobacteria	704	90
Gammaproteobacteria	20780	517
Actinobacteria	22726	1029
Bacteroidetes	30373	660
Firmicutes	10441	346
Planctomycetes	2246	212
Chloroflexi	1383	118
Gemmatimonadetes	144	32
Acidobacteria	379	111
Other	1199	573
SUM	179560	6218

High Low

Figure S2. The proportional distributions of sequences and derived OTUs in each bacterial phylum detected in all straw samples.

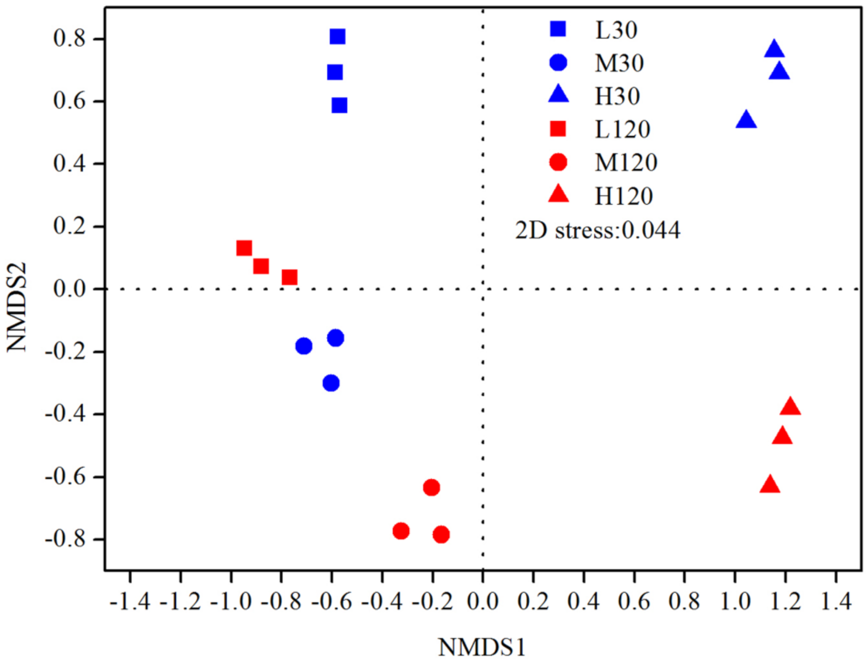

Figure S3. Non-metric multidimensional scaling (NMDS) of the bacterial communities based on Jaccard distances and symbols coded by treatments. L30: low temperature (15 ℃) on day 30 (early stage of decomposition); M30: moderate temperature (25 ℃) on day 30; H30: high temperature (35 ℃) on day 30; L120: low temperature on day 120 (late stage of decomposition); M120: moderate temperature on day 120; H120: high temperature on day 120.

Table S1. List of straw samples and corresponding barcodes used in this study.

Samples	Barcode
L30-1	ACGACACGTAT
L30-2	ACGCGTCTAGT
L30-3	ACGTACACACT
M30-1	AGTACGAGAGT
M30-2	AGTACTACTAT
M30-3	AGTAGACGTCT
H30-1	CAGTACGTACT
H30-2	ACTCACTAGCT
H30-3	ACTCTATATAT
L120-1	AGCGCACGAGT
L120-2	AGCGTGTGCGT
L120-3	AGCTAGATACT
M120-1	ATCGCTCACGT
M120-2	ATCGTCAGTCT
M120-3	ATCTCTCGTAT
H120-1	CACGCGAGTCT
H120-2	CACGCTACGAT
H120-3	CACGTGTATAT

L30: low temperature (15 ℃) on day 30 (early stage of decomposition); M30: moderate temperature (25 ℃) on day 30; H30: high temperature (35 ℃) on day 30; L120: low temperature on day 120 (late stage of decomposition); M120: moderate temperature on day 120; H120: high temperature on day 120.

Table S2. Number of sequences and OTUs measured by QIIME processing in each sample.

	Count	
	Sequences	OTUs
L30-1	6573	492
L30-2	2920	409
L30-3	3664	411
M30-1	1100	272
M30-2	4525	712
M30-3	2236	409
H30-1	1243	158
H30-2	2028	184
H30-3	1432	181
L120-1	26906	1880
L120-2	32910	1370
L120-3	33987	1393
M120-1	20903	1582
M120-2	15678	1340
M120-3	12089	1170
H120-1	5574	685
H120-2	3226	497
H120-3	2566	455

L30: low temperature (15 ℃) on day 30 (early stage of decomposition); M30: moderate temperature (25 ℃) on day 30; H30: high temperature (35 ℃) on day 30; L120: low temperature on day 120 (late stage of decomposition); M120: moderate temperature on day 120; H120: high temperature on day 120.

Table S3. The factors that significant correlated with bacterial communities of decomposing wheat straw were list below. The correlations (*r*) and significance (*P*) were determined by Mantel analysis between the variables and the bacterial community composition.

Variable	*r*	*P*
pH	0.513	0.001
DON	0.591	0.001
MBC	0.591	0.001
Straw C	0.284	0.004
Straw N	0.589	0.001
Straw P	0.475	0.001

Table S4. Direct and indirect effects of pathways of responses relative to bacterial community by path analysis.

Response	Direct effect	Indirect effect		
		Temperature	Soil properties	Straw properties
Temperature	-0.081	--	0.142	-0.130
Soil properties	1.043	-0.011	--	0.014
Straw properties	0.179	0.059	0.083	--
